# Prevalence of Helminthic Infections in the Gastrointestinal Tract of Cattle in Mazandaran Province (Northern Iran)

**DOI:** 10.1155/2022/7424647

**Published:** 2022-06-07

**Authors:** Nasrollah Vahedi Nouri, Reza Rahmatian, Alireza Salehi

**Affiliations:** ^1^Animal Parasitic Disease Research Department, Razi Vaccine and Serum Research Institute, Agricultural Research, Education and Extension Organization (AREEO), Karaj, Iran; ^2^Department of Animal Science, Agricultural and Natural Resources Research Center of Mazandaran, (AREEO), Sari, Iran; ^3^Department of Veterinary Parasitology, Babol Branch, Islamic Azad University, Mazandaran, Iran

## Abstract

Ruminant parasites are found in all parts of the world, including the tropical and subtropical regions. Mazandaran province (northern Iran) is one of the areas prone to the activity of gastrointestinal parasites in ruminants. This study was performed in 2020, in order to identify common parasites of cattle gastrointestinal tract, the percentage of the infection, the effect of seasons, and livestock on the amount of infection and determine the severity of infection. In this study, seven genera of gastrointestinal parasites including *Strongyloides*, *Haemonchus*, *Ostertagia*, *Cooperia*, *Trichostrongylus*, *Oesophagostomum*, and *Chabertia* were identified among 240 cattle. Overall, 34.58% of cows were infected with different kinds of parasites. Based on the average percentage of livestock infected with gastrointestinal parasites across different seasons, winter had the lowest percentage (18.33%) compared to other seasons, which has a statistically significant difference (*P* < 0.05). Also, we observe that the female cattle's infection rate, in comparison with males, had no significant difference (*P* < 0.05). Moreover, 67.17% of the cattle had a moderate level of infection. Despite the fact that farmers use antiparasitic drugs, helminthic infections in cattle are still high. In conclusion, the lack of proper implementation of helminthic infection control programs as well as antiparasitic drug resistance in this area can be a key element for the high prevalence of livestock helminthic infection in these areas.

## 1. Introduction

Cattle are used as the main part of Iran's livestock industry. The results of previous research conducted in Iran showed that ruminant domestic animals are infected with various types of parasites, including gastrointestinal parasites [1–3]. Despite its growth in the last few years, industrial livestock breeding is still mainly based on pastures, which exposes animals to a variety of diseases, such as parasitic diseases. Anorexia, diarrhea, growth retardation, and significant economic damage are all symptoms of gastrointestinal helminthic infections, which are caused by many types of parasites [4]. Daily milk output is lowered by 1 kg in cows with gastrointestinal parasites, according to research. In terms of economics, gastrointestinal parasite infection has been linked to a 0.15% drop in profitability and 50% weight loss [5]. Furthermore, reproductive efficiency is harmed [6]. Worms, on the other hand, infect calves and cause severe parasitic gastroenteritis. Nematodes of the *Strongylida* order, in particular, can cause serious difficulties in the tropics [7]. While immature worms (larvae) cause bacterial and fungal infections in animals by migrating to various tissues in the body, some adult worms produce toxins that cause indirect anemia in animals by reaching bloodstream and destructing RBCs [8]. Nematode infection kills animals in tropical and subtropical areas, particularly in regions where livestock feed is inadequate or infected [9]. Trematodes, cestodes, and protozoa are other parasites that affect ruminants, with some of them being common in both humans and animals and posing a public health risk. In the field of helminthic infections of nematodes, cestodes, and trematodes of the gastrointestinal tract of Iranian ruminants, relatively a lot of research has been done. However, geographical location and season have a strong impact on the occurrence of these many infections. The life cycle of parasites is also influenced by climatic factors, such as ambient temperature and rainfall patterns [10]. This is particularly important in Mazandaran province (northern Iran), where animal husbandry is a key element of the people's economy and social life. As a result, this study was carried out in Mazandaran province, in order to identify common parasites of cattle's gastrointestinal tract, determining the percentage of infection, the effect of seasons, and gender on the amount of infection and determining the severity of infection, based on epidemiological data. The goal of this observational study was to count the number of parasite eggs per gram of feces in order to assess the existence and kind of helminthic infection, as well as the intensity of that.

## 2. Materials and Methods

### 2.1. Study Area

**Figure 1:**
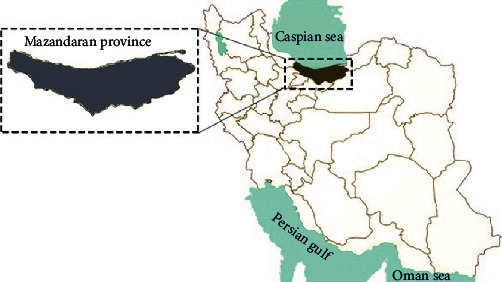
Location of Mazandaran province in the map of Iran.

Mazandaran is located in northern Iran, on the Caspian Sea's southern coast (Figure 1). This province, which is located at 36.23° N, 52.53° E and covers an area of 23833 square kilometers, accounts for 1.5 percent of Iran's total land area. Like the great wall, the Alborz mountain range has split Mazandaran into lowlands and mountains. Due to the sea, mountains, and forests, Mazandaran's climate is classified into two types: moderate humidity and mountainous. The presence of the Caspian Sea and the Alborz Mountains, as well as their proximity to each other, has resulted in a temperate and humid climate, with particularly hot and humid summers on the shore. Winters in these areas are moderate and humid, with only a few frosty days. For these reasons, animal husbandry is one of the most prominent occupations in this area due to the ideal geographical conditions [11].

### 2.2. Study Design

The status of traditional farms in Nowshahr, Amol, and Sari cities as sampling regions of Mazandaran province was determined in the course of this research in 2020 across four seasons, which included field and laboratory studies. Out of 240 samples gathered over the course of a year, 120 of them were male, and the rest were female. A farm with more than 200 cows was chosen in each area, and only farms that had the same management for their past year were considered. The animals' gender was recorded on the necessary forms. Sampling was done at the beginning and middle of each season. One out of every ten animals was examined in a 10% intensity sampling with a regular random sequence. Each season, 60 samples were gathered, half of which were female and the other half were male. As a result, the total number of samples gathered across the four seasons was 240. Approximately 30 to 50 grams of excrement was collected straight from the animal's rectum and kept into special sample collection containers individually. It should be noted that Albendazole, an antiparasitic drug, was used by farmers twice a year at the beginning of spring and autumn in the region annually. So to be more accurate, those farms were avoided. To avoid the hatching of larvae eggs, the samples were evaluated in the laboratory 2 hours after sampling. The Clayton-Lane method was used to count the amount of parasite eggs in the feces in this study [12]. During the four seasons, differences in infection rates between males and females were observed, and the percentage of parasite species on the animals was calculated.

### 2.3. Sampling

After gathering enough samples, approximately mix 3 grams of excrement with 42 ml of regular water. Then, using a glass ball and sandpaper, stir the mixture in a glass until it is homogenous. A 100-mesh sieve was used to filter the prepared mixture in the beaker. After stirring, a portion of the filtered liquid was placed into a Clayton-Lane centrifuge tube with a volume of 15 ml. The liquid was centrifuged for 2 minutes at 1500 rpm. Water was used to perform the washing process numerous times. The upper liquid, which contained grease and colors, was discarded after washing, and the remaining sediment from the bottom of the tube was removed by flipping to the sediment region at the tube's end. Fill the tube halfway with saturated sodium chloride water solution, and gently mix it with the thumb as the lid several times. After that, the tube was filled to the brim with saturated solutions and centrifuged for 2 minutes at 1000 rpm after laminating the tubes. The lamellae were then placed on a slide and examined under a microscope on a regular basis. The number of eggs counted below the lamellae represents the number of eggs per gram of excrement since the total volume of the initial mixture is 45 ml, and the volume of the centrifuge tube is 15 ml. At this point, 1/6 of the eggs were still in the silt at the bottom of the tube; thus, the number of eggs mentioned below the lamellar must be increased. The coefficient of adjustment of fecal concentration (normal feces: 1, semiloose: 1.5, loose: 2, and diarrhea: 3) should also be included in the calculation for the definitive determination of the number of eggs per gram of feces. Heavy eggs, such as *Fasciola*, *Dicrocoelium*, and *Amphistomes* eggs, and larvae of pulmonary worms were found in the sediment at the bottom of the tube after this stage, as well as some light eggs and *Trichuris* eggs. The prior solution was removed first, followed by the sediment from the tube's bottom, but instead of sodium chloride alone, a saturated room-temperature solution of zinc chloride and sodium chloride was used to float the trematode eggs [12]. In the fecal test, the eggs of nematodes (*Haemonchus*, *Ostertagia*, *Cooperia*, *Trichostrongylus*, *Oesophagostomum*, and *Chabertia*) whose type can be difficult to differentiate from the appearance of eggs were tallied together as *strongylid*.

### 2.4. Stool Culture

A fecal culture test was performed to create L3 larvae in order to determine the sort of worm eggs that are inseparable from the look of the feces. The Berman method was used to separate the larvae from the cultured feces. A spatula was used to finely chop about 20 grams of excrement. It was then poured into a screw-top glass with a large opening. For dried stools, a small amount of water was added to equalize the moisture. Then, the cylindrical glass lid was perforated to allow air to pass through. For 5 to 7 days, the culture dish was kept at 25°C. A funnel with a plastic tube attached to one end and a clamp attached to the other end was placed on a base and filled to the brim with lukewarm liquid (maximum 30°C). A sterilized gauze was placed on top of the funnel brim. About 3 grams of cultured feces was weighed and deposited on the sterilized gauze inside the funnel without crushing. Water droplets on the glass wall above the cultivated feces usually catch nematode larvae. As a result, the larvae could be recognized by collecting these water droplets. However, after soaking the feces, some of the nematode larvae would be deposited and collected in the Berman tube after passing through the Gauze or mesh pores behind the clamp due to the lack of surface tension inside the funnel tube because of gravity. The observation was also carried out by opening the clamp and placing 1-2 drops of the clamp's solution onto the slide. 2-3 drops of Lugol's iodine were added to the required liquid to determine the gender of nematode larvae precisely [12]. Finally, the third-stage larvae (L3) were identified [13].

### 2.5. Data Analysis

Three levels of contamination were determined based on the number of eggs per gram of fecal sample: low contamination (the average number is less than 100 eggs per gram of fecal sample), moderate contamination (the average number of eggs is between 100 and 500 per gram of fecal sample), and high contamination (the average number is greater than 500 eggs per gram of fecal sample). The findings were analyzed using the Chi-square test based on [14].

## 3. Result

In this study, each season 60 samples were gathered, totaling 240 samples over the course of a year. The findings of this study are organized into four categories.

### 3.1. Seasonal Findings

According to the findings, the average percentage of the cows with gastrointestinal parasites was (34.58%). Also, the average percentage of cows infected with gastrointestinal parasites in different seasons showed that the lowest level of infection was at winter (18.33%) when compared to other seasons, which was at a significantly lower proportion (*P* < 0.05) (Table 1).

### 3.2. Gender Comparison

One of the most important aspects of this study was the level of a parasite infection that varies by the animal's gender. The findings revealed that helminthic infection in the female cattle was not significantly different from the male cattle. The average frequency of gastrointestinal parasites in females and males were 34.28% and 34.63%, respectively.

### 3.3. Types of Parasites

**Figure 2:**
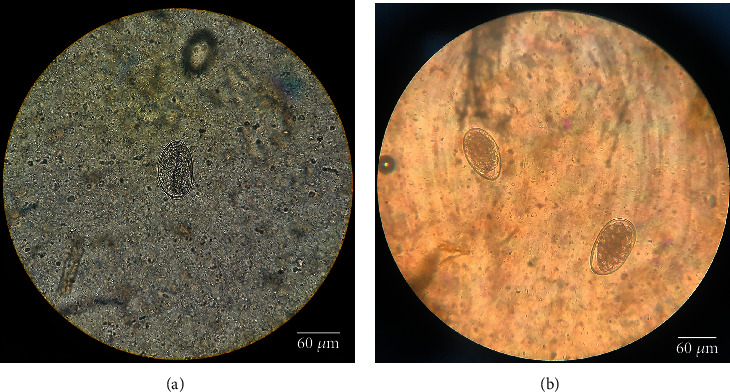
Eggs of parasites of cattle: (a) *Strongyloides* sp. (Ellipsoid, 40–85 *μ*m in length, with a thin wall containing the first-stage larva). (b) *Strongylid* (approximately 80 *μ*m long, thin-shelled, broad ellipse, barrel-shaped side walls, and blastomeres present). Magnification 40X.

**Figure 3:**
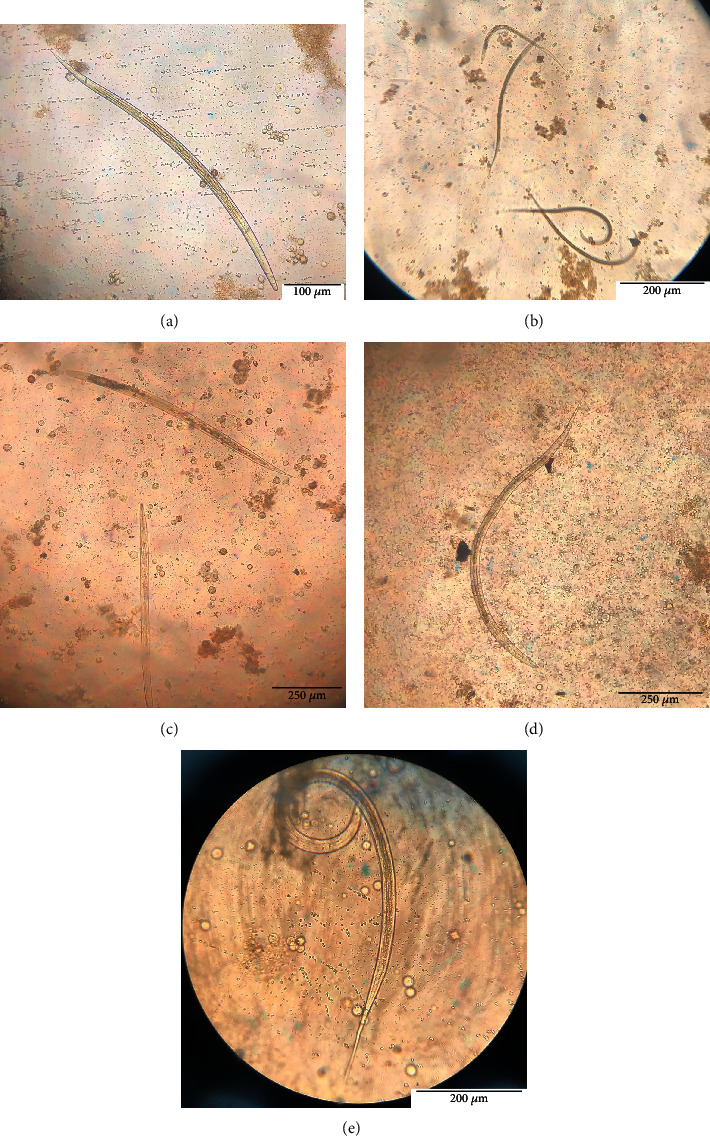
Third-stage larvae (L_3_) after fecal culture: (a) *Trichostrongylus* sp. (head rounded, short-sized larva, tail of sheath short, and bearing one or two tuberosities), (b) *Chabertia* sp. (tail of sheath very long, larva of medium size, 24-32 square gut cells, and lumen of gut straight), (c) *Cooperia* sp. (head squared with two refractile oval bodies at the anterior end of the esophagus, large-sized larva, and medium-length sheath that tapers to fine point), (d) *Haemonchus* sp. (head rounded, medium-sized larva with medium length, and kinked sheath tail), (e) *Oesophagostomum* sp. (rounded head, medium-sized larva, long thin sheath tail, and 16–24 triangular intestinal cells). Magnification 40X.

The parasites that were found in the cattle of Mazandaran province were examined immediately after sampling (Table 2) (Figures 2(a) and 2(b)). Due to their inseparability, some of the eggs were analyzed and then were classified as *strongylid*; hence, a stool culture test was done to determine their type. In the investigated cows, the fecal culture revealed the presence of the following species: *Haemonchus*, *Ostertagia*, *Cooperia*, *Trichostrongylus*, *Oesophagostomum*, and *Chabertia* (Tables 2–4) (Figures 3(a)–3(e)).

### 3.4. Frequency

The number of parasite eggs per gram of fecal samples was determined in this observational study to establish the degree of helminthic infection in cattle. The percentage of cows with low infection intensity was 32.83%, whereas the percentage of cows with moderate infection intensity was 67.17% which was statistically significant (*P* < 0.05). The severity of low infection intensity in different seasons of the year, on the other hand, indicates that winter significantly has the most severity of low infection intensity among all seasons(*P* < 0.01). However, winter significantly has the lowest average level of moderate infection intensity between seasons (*P* < 0.01) (Table 4).

## 4. Discussion

According to our findings, the average percentage of cows infected with gastrointestinal parasites was 34.58%. This rate of infection is impressive when considering that all of the investigated animals were given a broad-spectrum antiparasitic medicine. The average percentage of gastrointestinal parasite infection in cows in different seasons demonstrates that the winter has the lowest level of infection, with a statistically significant difference from the other seasons (Table 1). The cause of the comparatively higher degree of infection of cattle in spring, summer, and autumn compared to winter, given the geographical characteristics of Mazandaran province, would be discussed from two perspectives. The first reason is that in winter, the gastrointestinal parasites in cattle significantly are reduced due to the cold weather, inaccessibility to open pastures and grazing, and livestock presence in enclosed areas. The second reason is that as spring arrives, the relative temperature rises, and the humidity level decreases because of the rains. These conditions last until late autumn, with an increased probability of being infected by the survived larvae, as well as the free grazing of cattle in pastures, which increases the average proportion of helminthic infection in both sheep and cats. This finding is consistent with the earlier research [15–17]. All environmental factors such as the humidity, temperature, rainfall, sunlight, wind, weather patterns, and forestation play a specific role in the epidemiology of ruminant parasite infections [18]. Furthermore, based on the findings of this study, the average rate of gastrointestinal parasites in female and male cows was estimated to be 34.28% and 34.63%. Females did not differ significantly from the males in terms of parasite infection. This may be caused by an increased level of both prolactin and progesterone hormones in the blood due to the stress during pregnancy, muscle weakness following childbirth, and nursing. Consequently, weakened female immune systems would lead to an increased infection rate [19, 20]. Previous studies' results support the findings of this investigation as well [18, 21, 22].

Under unfavorable environmental conditions (usually at the end of the grazing season and at the same time as the onset of winter), the larvae of some parasites enter the hypobiosis stage in the ruminant's digestive tract. These arrested larvae usually resume their growth with the adaptation of environmental conditions such as the beginning of spring, so the diversity of the parasites in winter is less than in other seasons [23]. Consistent with the results of previous studies [24–26], with the onset of spring, and the use of pastures for the livestock, followed by an increase in the temperature, the required conditions for the growth of larvae in the eggs would be provided, and the eggs would hatch; thus, the infection rate of cattle will increase. This situation (increased temperature with rainfall) lasts until the end of November. This trend increases the parasitic infection intensity in the summer and autumn, so that in autumn, the amount of infection is more than that of other seasons (Table 3).

Overall, seven genera of gastrointestinal parasites were found in cows in Mazandaran province as part of this study. Gastrointestinal nematodes were among the parasites identified in this investigation. *Haemonchus*, *Ostertagia*, *Cooperia*, *Trichostrongylus*, *Oesophagostomum*, and *Chabertia* are among the nematode genera that were identified. Frequently, more than one parasite genus was detected from a single host in this study, which is similar to the results of previous studies [17, 27, 28]. *Trichostrongylus*, *Chabertia*, and *Oesophagostomum* were the most prevalent parasites in this study based on fecal culture. When the results of this study were compared to those of earlier studies, it was clear that the parasite diversity in the cattle of Mazandaran province is relatively substantial [29, 30]. Our findings show that the low-intensity infection was 32.82%, moderate-intensity infection was 67.17%, and high-intensity infection was not observed. This usually happens because of the farmers' widespread usage of livestock antiparasitic medicines. When the intensity of infection in different seasons of the year was compared together, a significant difference was observed (*P* < 0.05). The results showed that throughout the three seasons of spring, summer, and autumn, when cattle breeding is at its peak level, the average intensity of infection is much lower than the severity of infection during the winter (*P* < 0.01). Since the animals were more sheltered and under nutritional control in the winter, the average level of low-intensity infection was significantly higher than the total average of infection intensity (Table 4).

## 5. Conclusion

Our study demonstrates that despite the wide usage of broad-spectrum antiparasitic drugs by farmers in recent years, helminthic infections are still present in the animals. Due to the significant presence of helminthic infections in cattle in Mazandaran province, this situation reveals the farmers' failure to properly execute helminthic infection management programs in this region. Therefore, as a response, it is critical to give awareness on the proper use of antiparasitic treatments, as well as the economic losses caused by them and the parasite's life cycle. On the other hand, antiparasitic drug resistance should not be underestimated. Besides all previous factors, rainfall, humidity, and temperature are all important climatic elements in parasitic infection. Farmers should establish a parasite infection control plan based on the seasonal prevalence variations, age groups, farmland management, dietary modification, proper use of antiparasitic medications, and breeding programs. Cattle should not be left to graze on a pasture constantly, and pasture rotation (intermittent grazing) should be considered. Finally, at the beginning and end of the rainy season, veterinarians should advise and persuade farmers to use broad-spectrum antiparasitic medications based on the evidences and tests.

## Figures and Tables

**Table 1:** The average percentage of cows having gastrointestinal parasites varies in different seasons.

Spring	Summer	Autumn	Winter	Chi-square
35.00%	40.00%	45.00%	18.33%	15.37^∗^

^∗^ means significant difference at 0.05 level (*P* < 0.05).

**Table 2:** Number (percentage) of observed gastrointestinal parasites.

Genus	Spring	Summer	Autumn	Winter
*Strongyloides*	10 (16.67%)	0	0	0
*Strongylid*	21 (35%)	24 (40%)	27 (45%)	11 (18.33%)

**Table 3:** Seasonal variation of parasites.

Genus	Spring	Summer	Autumn	Winter
*Strongyloides*	10 (16.67%)	0	0	0
*Haemonchus*	3 (3.3%)	3 (3.3%)	7 (11.6%)	1 (1.6%)
*Ostertagia*	2 (3%)	4 (6.6%)	5 (8.3%)	0
*Cooperia*	2 (3%)	4 (6.6%)	4 (6.6%)	7 (11.6%)
*Trichostrongylus*	21 (35%)	24 (40%)	27 (45%)	11 (18.33%)
*Oesophagostomum*	5 (8.3%)	5 (8.3%)	7 (11.6%)	0
*Chabertia*	7 (11.6%)	11 (18.33%)	(15%)	(15%)

**Table 4:** Comparison of the average percentage of cows infected at different seasons of the year.

Season	Low infection intensity	Moderate infection intensity	High infection intensity	Chi-square
Spring	14.29%	85.71%	0	51.84^∗∗^
Summer	16.67%	83.33%	0	43.56^∗∗^
Autumn	18.52%	81.48%	0	40.16^∗∗^
Winter	81.82%	18.18%	0	40.96^∗∗^
Total	32.83%	67.17%	0	11.56^∗^

^∗∗^ means a significant difference in each row (*P* < 0.01).

## Data Availability

Data are available on request from the corresponding author.
